# Vaccine Acceptance and Its Influencing Factors: An Online Cross-Sectional Study among International College Students Studying in China

**DOI:** 10.3390/vaccines9060585

**Published:** 2021-06-02

**Authors:** Anita Nyarkoa Walker, Ting Zhang, Xue-Qing Peng, Jin-Jin Ge, Hai Gu, Hua You

**Affiliations:** 1 School of Public Health, Nanjing Medical University, Nanjing 211166, China; walkeranita30@gmail.com (A.N.W.); Pengxueqing1230@163.com (X.-Q.P.); gejjin@163.com (J.-J.G.); 2 School of Public Administration, Zhejiang Gongshang University, Hangzhou 310018, China; zhting1978@sina.com; 3 School of Government, Nanjing University, Nanjing 210093, China; ghai1008@nju.edu.cn

**Keywords:** vaccine acceptance, health behavior theory, cross-sectional study, China

## Abstract

Background: With the continuous large-scale development of the COVID-19 vaccine, the acceptance of vaccination and its influencing factors at the individual level have become crucial to stemming the pandemic. This study aims to explore the factors that influence the acceptance of the COVID-19 vaccine among international college students. Methods: The target population constituted international students pursuing various degrees in Jiangsu Province through an online cross-sectional study. A cluster random sampling was performed using a self-administered questionnaire. The Health Belief Model and Knowledge, Attitude/Beliefs, and Practice Theory served as the underlying theories to understanding the factors that influence vaccine acceptance. Results: We received 330 responses. About 36.4% intended to accept the vaccine. The acceptance varied across respondents’ place of residence, program of study, continent of origin, knowledge, susceptibility, severity, benefits, and cues to action (*p* < 0.05). A multivariable logistics regression revealed cues to action (*p* < 0.001), the perception of COVID-19 vaccination benefits (*p* = 0.002), and the perception of barriers (*p* < 0.001) that were associated with vaccine acceptance. Conclusions: The acceptance of the COVID-19 vaccine was low among international students. The correct and comprehensive beliefs of the target groups regarding the benefits and barriers of the vaccination must be raised. Various effective social strategies must be adopted to trigger the intention of COVID-19 vaccination. The study findings will inform the decisions of public health campaigners, aimed at reducing vaccine hesitation when the COVID-19 vaccine is widely available.

## 1. Introduction

Coronavirus disease (2019), caused by SARS-CoV-2, is an RNA virus that belongs to the family Coronaviruses. COVID-19 is a highly infectious disease with the following symptoms: fever, dry cough, fatigue, myalgia, and dyspnea [1,2]. In December 2019, researchers initially traced the root of the disease to a seafood market in Hubei Province, China [3], but it has since spread to 222 countries and territories, with 128.8 million confirmed cases. Out of this total, over 73 million have recovered from the disease, with 2.8 million deaths recorded throughout the world (as of 1 April 2021). The Coronavirus pandemic has resulted in severe implications on various economies across the globe. For instance, a host of countries have recorded negative trends in growth, thereby affecting the world’s economy. A 5.2% decline was observed in the global gross domestic product (GDP) [4], increasing mortality and placing restrictions on the normal life of individuals.

Hence, vaccination, which is one of the avenues to prevent susceptible and infectious diseases, is required. For instance, influenza vaccination is one of the most convenient ways to control seasonal influenza, among other related diseases [5]. Vaccines have been identified as one of the most efficient ways of eluding further COVID-19 outbreaks. Additionally, the response to COVID-19 has been estimated to have long-term success when a herd immunity of 52–82% is achieved [6], but this can only be achieved through widespread vaccination. This fends for individuals who have been vaccinated and, also, induces indirect protection (herd immunity) for the entire community by preventing person-to-person transmission [7].

However, a successful vaccination program is largely affected by the rate of acceptance, which is unsatisfactory across the globe. Kwok et al. (2020) reported that about 70% of the population they surveyed accepted to take the COVID-19 vaccine upon its readiness [8]. About 26% of adults from seven European countries like the UK suggested they were unsure or unwilling to receive a COVID-19 vaccine when made available [9]. Likewise, only a quarter of the French [10] and the US [11] reported their readiness to accept the COVID-19 vaccine. Similar to this, the young population, specifically students, have shown varying acceptance rates, from low to high. For instance, 53% of medical students in Southeast Michigan [12], 86% of university students in Italy [13], 45.3% of nursing students in Northeast University [14], and 91.64% of college students [15] accepted to take the COVID-19 vaccine

Various theories have been proposed to explain the behavior of people during disease outbreaks. Commonly used theoretical frameworks entail the Health Belief Model (HBM) and Knowledge, Attitude/Beliefs and Practice Theory (KABP). The HBM consists of constructs like perceived susceptibility, perceived severity, perceived benefits, perceived barriers, and cues to action [16]. Perceived susceptibility implies the belief of the possibility of an infection, while perceived severity is the negative consequence of being infected with the disease. Perceived benefits concerning vaccination refers to the individual’s beliefs of the importance and essence of being vaccinated. Inversely, perceived barriers are the constraints related to vaccination due to misinformation, psychological, physical, or monetary factors. Cues to action include information, people, and other activities that affect an individual’s vaccination status [16]. Different studies have used the HBM to ascertain the perceived benefits in relation to the vaccine decreasing the probability of an infection and making people less worried about contracting the disease [17]; a perceived susceptibility linked to the notion of a high risk of infection [18] and perceived barriers linked to a limited belief or mistrust in the efficacy of the vaccine are some of the factors that affect vaccine acceptance. Likewise, vaccination knowledge is the individual level of awareness or sensitization regarding vaccinations. The attitudes/beliefs refer to the feelings people have, as well as the predetermined ideas one harbors towards a vaccine, while the practice involves putting into action the knowledge, beliefs, and other relevant factors that affect vaccination [19]. A low level of knowledge about the COVID-19 vaccine has been reported to influence the rate of vaccine acceptance [20]. Similarly, undesirable beliefs about the COVID-19 vaccine due to lack of awareness among the Middle East population have also been identified as a cause of vaccine refusal [21].

The young population perceive themselves not to be susceptible to COVID-19, hence their inability to adhere to the COVID-19 safety protocols [22]. However, recent cases of asymptomatic transmissions being recorded later depict severe symptoms among the young population. Thus, they transmit the virus unknowingly without the initial symptoms being detected among them [23]; hence, vaccinating such population is very crucial. Additionally, studies that focus on students outside their home country are limited. Considering the diversity of nationalities and cultural backgrounds of various students enrolled in various programs, perceptions and behaviors regarding vaccination could differ. Therefore, it is necessary to know their perceptions towards vaccine acceptance. In addition, a global perspective concerning the influencing factors of the vaccine can be obtained from their responses. Students are a good target for educational campaigns, since they are still on a learning curve; hence, their attitudes/beliefs at this stage can be influenced positively. Presently, understanding their perspective about the COVID-19 vaccine will be essential for appropriate immunization planning response and management strategies against the COVID-19 pandemic. We seek to use health behavior theories to determine the factors that are associated with COVID-19 vaccine acceptance.

## 2. Materials and Methods

### 2.1. Study Design, Population, and Sampling

We undertook an online cross-sectional survey using a questionnaire to identify the factors influencing the acceptance of the COVID-19 vaccine among International students pursuing various degrees in Jiangsu Province of China. Cluster random sampling was used in the survey. Seven universities with international students were randomly selected in Jiangsu Province. Two classes were randomly selected from the selected universities. The online questionnaire was distributed by volunteers of the selected classes in each university via WeChat social group platforms. However, slow and limited access due to Internet services on mobile phones resulted in the failure of 25 students among the sample size not being able to respond to the questionnaire. Hence, 330 (93%) students were enrolled and successfully completed the survey. The survey was performed from 17 March to 26 March 2021. The questionnaire could only be accessed once from the same WeChat account. Participation was voluntary, with no incentive. This study was anonymous, as no information concerning personal identification (confidentiality) was collected. A duration of five minutes could be used to complete the survey. Participant’s completion of the questionnaire was regarded as consent for participation.

### 2.2. Measures

Our questionnaire was constructed by reviewing similar works on vaccine acceptance [17,18,24,25] and information from the CDC and other authoritative websites [24,25,26]. It consisted of sociodemographic characteristics (age, gender, current place of residence, educational level, program of study, continent of origin (refers to the origin of respondents), general health status, chronic disease history, and average monthly expenses); knowledge concerning general vaccination and the COVID-19 vaccine (14 questions); and the constructs of the Health Belief Model, including susceptibility (5 questions), severity (5 questions), benefits of the COVID-19 vaccine (5 questions), barriers of the COVID-19 vaccine (5 questions), cues to action (5 questions), and vaccine acceptance (5 questions). Most of the questions were closed-ended with provided responses, except for a few. For instance, questions that related to the main side effects were semi-open and semi-closed. Knowledge-related questions had response options of Yes, No, or I don’t know. Regarding the main side effects, recommended dose interval, and recommended groups required to receive the vaccine, possible responses were given to respondents to select what they deemed fit. Similarly, all the questions in the construct of the HBM and vaccine acceptance were measured on a five-point Likert scale, strongly agree to strongly disagree. The questionnaire was piloted among 30 students from different universities to find out if the questions were clear and understandable. All the responses were reviewed before the final questionnaire was sent out. To test for the internal consistency and reliability of our questionnaire, we performed Cronbach’s alpha for the questions of each construct of the HBM (please refer to the Supplementary Materials for the results).

### 2.3. Statistical Analysis

The acceptance of the COVID-19 vaccine was our dependent variable, while our independent variables were the demographic variables, health beliefs, and knowledge. The health belief constructs and acceptance, which were measured on a five-point Likert scale of strongly agree or agree, was given a score of 1, while the rest were recorded as 0. However, two questions regarding acceptance: “I still have some concerns so I don’t want to get the COVID-19 vaccine right away” and “I’m still hesitant about the COVID-19 vaccine” were reverse-coded; hence, disagree or strongly disagree were recognized as 1. Next, all the questions were summed for each part, and a score of 3 or more was recognized to have that belief (such as susceptibility, among others.) or attitude (acceptance), while the opposite was given a score of ≤2. In determining the knowledge scores, responses were recorded as 0 for incorrect responses and 1 for correct responses. All the correct answers were summed up and grouped into two “knowledge Yes” and “knowledge No”. Respondents with a score >7 were given “knowledge Yes”, while those who scored ≤7 were given “Knowledge No”. Likewise, Crosstabs were used to know how the acceptance varied across the demographic variables, the constructs of the Health Belief Model, and knowledge. A multivariable logistic regression was conducted by using the demographic variables, health beliefs, and knowledge as the independent variables, while COVID-19 vaccine acceptance was regarded as a dependent variable. We analyzed our data using SPSS version 25.0 (SPSS Inc., Chicago, IL, USA), with the statistical level set at *p* < 0.05 (two-sided).

## 3. Results

### 3.1. Sociodemographic Characteristics

Out of the 330 responses received, more than half of the respondents were males, within the 18–25 age group. They were primarily undergraduates who currently reside in China. Half of them were African, whilst 45.8% were Asian. The respondents revealed they were healthy (94.8%). The majority of the respondents’ average monthly expenditures ranged between 1000 and 3000 CNY (152.6 and 457.8 USD) (Table 1).

### 3.2. Knowledge

About 67% of our respondents had adequate knowledge about general vaccinations and the COVID-19 vaccine (Supplementary Table S1). However, 55.2% of the respondents believed vaccinations could make people sterile, 73.3% and 72.7% were not aware of the common side effects of the COVID-19 vaccine, coupled with that individuals currently infected with COVID-19 should not vaccinate, respectively (Table 2).

### 3.3. Health Beliefs and Vaccine Acceptance

#### 3.3.1. Perceived Susceptibility

Our respondent’s perceptions of susceptibility toward COVID-19 were low, with 38.8% reporting as susceptible (Supplementary Table S1). About 48.2% agreed to infection possibility, 42.8% were concerned about those around them being infected, while only 15.7% reported a greater chance of being infected (Table 3).

#### 3.3.2. Perceived Severity

Generally, about 50.9% of our respondents saw COVID-19 as severe (Supplementary Table S1), with 75.2% seeing the complications of COVID-19 as severe, 61.3% perceived the infectivity to be high, 55.2% said it can cause serious health damage, while 24% perceived COVID-19 to be a death threat (Table 3).

#### 3.3.3. Perceived Benefits

The majority of respondents (64.5%) perceived the COVID-19 vaccine to be beneficial (Supplementary Table S1). They reported that the vaccination reduces the chance of infection (66.9%) and prevents complications (56.7%). Hence, it is of great importance to be vaccinated (62.9%) (Table 3).

#### 3.3.4. Perceived Barriers

More than half of our respondents were hindered by barriers (58.5%) (Supplementary Table S1). They were concerned about the COVID-19 vaccine interrupting their normal life (58.5%), safety (66.0%), and the authenticity of the vaccine (56.7%). Nevertheless, only 36.7% were worried about the side effects, with 39.7% being concerned about the affordability of the vaccine (Table 3).

#### 3.3.5. Cues to Action

Approximately 51.2% were influenced by cues (Supplementary Table S1), where 68.1% intended to vaccinate when they got adequate information the government readiness to give free vaccines (50.9%). However, only 30% reported being encouraged by family and friends to vaccinate. Similarly, 39.7% agreed to vaccinate when they saw others vaccinated (Table 3).

#### 3.3.6. Vaccine Acceptance

The proportion of respondents accepting of the COVID-19 vaccine was 36.4% (Table 4). About 31.5% of the participants still had some concerns, whilst 30.6% were still hesitant in taking or accepting the COVID-19 vaccine (Table 3).

### 3.4. Results of the Univariate Analysis

A total of 36.4% of the 330 respondents strongly agreed/agreed to be vaccinated. Columns four and five of Table 4 show the responses of strongly agree/agree versus neutral/disagree/strongly disagree toward vaccination acceptance based on demographic characteristics, along with the constructs of the health belief model. The univariate analysis revealed that a higher proportion of respondents studying medical-related majors (40.9%) currently residing outside China (50.0%) strongly agreed/agreed to accept the COVID-19 vaccine. Similarly, a higher proportion of respondents who were knowledgeable (43.7%), susceptible (49.2%), perceived COVID-19 to be severe (42.9%), saw the COVID vaccine to be beneficial (50.2%), and were influenced by cues (60.4%) strongly agreed/agreed to accept the COVID-19 vaccine (Table 4).

### 3.5. Results of Multivariate Analysis

A multi-logistic regression analysis revealed that the cues to action (*p* < 0.001) predicted vaccine acceptance the most, followed by benefits of the COVID-19 vaccine (*p* = 0.002), while barriers to receiving the COVID-19 vaccine hindered the vaccine acceptance (*p* < 0.001). The perception of susceptibility, severity, and being knowledgeable were not significant (Table 5).

## 4. Discussion

This study was the first to explore the factors associated with COVID-19 vaccine acceptance using both HBM and KABP theories. The present study explored the views of international students studying in China as the key subjects of this research topic. About 36.4% of the respondents intended to accept the COVID-19 vaccine when made available, which is much lower than what was observed among students in Michigan [12], Italy [27], and the Northeast of the United States [15]. This rate of acceptance is very alarming, considering the nature of the population (i.e., a young population) that is still on a learning curve. However, a limited rate of vaccine acceptance has been established among the elite or inclined personnel [28]. This could be attributed to the elite in this context, who are skeptical and tend to criticize or rigorously scrutinize every situation to gain a comprehensive knowledge about the pros and cons of the situation at hand before accepting it. Additionally, individuals inclined to be study subjects of the subject matter are reported to be less convinced about the COVID-19 vaccines [28].

Our results found cues to action to be the highest predictor of vaccine acceptance. Cues in the form of adequate information and media promotion or sensitization were pivotal in our study. This is comparable to the study among medical students where the lack of information predicted hesitance toward the vaccinate [12]. Another study found a lack of trust and misinformation as the key factors that influenced the low rates of acceptance of the COVID-19 vaccine among the general population [29]. This reiterated the findings from the existing literature, which asserted that adequate information concerning vaccination is very crucial for the student population, as they will not accept anything without being informed about the benefits or essence of being vaccinated. Just like the work of Wong et al. (2020), the study participants were not influenced by the number of people vaccinating. Additionally, although the young population is normally influenced by peers and relatives, our participants were not influenced by their family, friends, or peers to vaccinate. This shows that getting vaccinated among this population is an individual decision or choice. Consequently, there is a need to facilitate more health promotion and sensitization programs to encourage them to vaccinate.

Perceived benefits to receiving the COVID-19 vaccine was the second-highest predictor of acceptance of the COVID-19 vaccine. The participants revealed their intentions of preventing and avoiding complications associated with the pandemic. These findings corroborated with the results of Wong et al. (2020), where their respondents’ reasons for accepting the vaccine were partly down to reducing the chances of being infected, because being vaccinated would make them less worried about the disease, which eventually increased their level of confidence and perception about the COVID-19 vaccine [17]. Again, the belief that vaccinating can normalize social lifestyles was another benefit, just like the work of Michaël Schwarzinger et al. (2021) [27]. Curiosity, exploration, and socialization are common among the young population; hence, they are more likely to vaccinate to enjoy their social lifestyles. Educational campaigns highlighting the benefits of the vaccination, like somewhat enjoying normalcy in one’s social life and the prevention of infections and complications, are required.

Numerous studies have enumerated barriers as one of the major negative predictors of vaccine acceptance, as reported in the present study. Like most studies, side effects, authenticity, and safety were the most reported barriers highlighted by the participants in the current study [30,31,32]. This could be attributed to the large number of vaccines produced within a short period, which lack adequate information in relation to the duration of protection and efficacy among a large population. Additionally, the newness of the COVID-19 vaccine could partly be another reason for the limited patronage. Most people are skeptical about the efficacy of new vaccines and have doubts about voluntarily or diversely accepting it, considering its span and effective usage in their current dispensation [33]. Our respondents were also concerned about the side effects of the vaccine interrupting their normal lives. These barriers can be reduced when college authorities give adequate information about the vaccine yet to be administered among interested participants. Additionally, for herd immunity to be achieved among this population, any misconceptions surrounding vaccines should be clarified by providing appropriate information to boost their confidence in accepting the COVID-19 vaccine.

The perceived susceptibility and severity of COVID-19 have been found by different studies as predictors of vaccine acceptance [18]. However, these variables were not significant in our study, although the odds ratio of these variables showed an increase in acceptance that was comparable to the work of Wong (2020) among the general population of Hong Kong. COVID-19 has been noted to be severe and susceptible mostly among the aged and those with immune-compromised systems [34]. Additionally, young populations, such as university students, usually see themselves as having a low risk against severe diseases. Research has revealed that individuals who see their risk and symptoms of infection as low and not severe, respectively, normally worry less about the disease, thereby feel no urgency or importance in taking the COVID-19 vaccine [31,35]. However, the Real-time Assessment of Community Transmission (REACT) study in the UK found that the 18–24-year-old group had the highest positivity rates, with 69% of those testing positive being asymptomatic [36]. Asymptomatic transmission of SARS-CoV-2 from the young population has been observed in the existing literature [37]. This calls for urgent need to raise more awareness on the increasing rate of asymptomatic transmissions within these groups to increase the rate of vaccine acceptance. Additionally, this population’s notion of not being prone gives more weight to the growing concerns for more education on vaccinations and asymptomatic transmissions.

A low level of knowledge has been one of the negative predictors of vaccinations, as revealed in existing studies [19]. Although 67% of the respondents’ knowledge concerning general vaccination was adequate, there were still knowledge gaps in relation to the individual recommended to vaccinate and the main side effects of the COVID-19 vaccine. This paucity of information can affect their intention to vaccinate. Limited information and misconceptions due to misinformation can affect an individual’s vaccination decision [33]. It is, however, not surprising that more than half of our respondents were of the opinion that vaccinations could make them sterile. A respondent reported that taking the COVID-19 vaccine can transform him or her into a zombie in the near future. For a learning population to have such misconceptions about vaccinations, proper education is required immediately and continuously to clarify or nullify these misconceptions. These acts would eventually trickle down and further educate participants’ peers and family members who have similar notions.

Despite the reasons explained above, there were several limitations in this study. First and foremost, this study used a self-reported questionnaire, which was subject to bias or mixed feelings among the respondents. Second, this was a cross-sectional study that provided a correlation explanation, but the causal relationship between vaccine acceptance, knowledge, and beliefs were not delved into. Third, our cut-off points used to classify the HBM constructs and knowledge were not referenced elsewhere, which may produce bias in the study results. Last, but not least, the scope of this study was limited to universities in Jiangsu Province; the results can be used as a reference but cannot be extrapolated to all international college students studying in China or other countries.

## 5. Conclusions

This study showed that 36.4% of our respondents intended to accept the vaccine against the Coronavirus disease (2019). The HBM can be used as basis for campaigns. In the strategy of improving vaccine acceptance, in addition to popularizing the relevant knowledge and the threat from infection, key interventions should be provided to enhance the benefits perception of vaccination and to reduce the barrier perceptions. Appropriate measures to increase the cues should be considered, since cues to action in social environments are found to be very critical promoters.

## Figures and Tables

**Table 1:** Demographic characteristics of the respondents (*n* = 330).

Variables		Frequency	Percentage
Gender			
	Male	180	54.5
	Female	150	45.5
Age			
	18–25	180	54.5
	26 and Above	150	45.5
Education			
	Undergraduate	172	52.1
	Postgraduate and above	158	47.9
Current Place of Residence			
	In China	192	58.2
	Outside China	138	41.8
Continent of origin			
	Europe	7	2.1
	Asia	151	45.8
	Africa	165	50.0
	America	3	0.9
	Oceania	4	1.2
Diagnosed with chronic disease			
	Yes	14	4.2
	No	316	95.8
General health status			
	Very good/good	330	94.8
	Fair/Bad	17	5.20
Average monthly expense			
	Less than 1000 CNY (152.6 USD)	102	30.9
	Between 1000 and 3000 CNY (152.6–457.8 USD)	188	57.0
	More than 3000 CNY (457.8 USD)	40	12.1

**Table 2:** Knowledge on general vaccinations and the COVID-19 vaccine.

Variable	Correct (%)	Wrong (%)
Vaccination is highly recommended to high-risk individuals (Individuals more vulnerable to the infection).	246 (74.5)	84 (25.5)
Can vaccination protect the people around you?	256 (77.6)	74 (22.4)
Do individuals with (a supposed) strong immune system need vaccination against diseases?	242 (73.3)	88 (26.7)
Is natural protection from being infected with the disease better than protection from vaccination?	231 (70.0)	99 (30.0)
Does vaccination make people sterile?	148 (44.8)	182 (55.2)
Vaccine-preventable diseases are not very dangerous; hence, there is no need to be vaccinated.	232 (70.3)	98 (29.7)
Vaccinations are not effective and do not prevent diseases.	262 (79.4)	68 (20.6)
The recommended dose interval of the COVID-19 vaccine.	196 (59.4)	134 (40.6)
The group of people recommended to get the COVID-19 vaccine.	73 (22.1)	257 (77.9)
The main side effects of the COVID-19 vaccine.	88 (26.7)	242 (73.3)
Should a person previously infected with COVID-19 still be vaccinated?	201 (60.9)	129 (39.1)
Should a person currently infected with COVID-19 still be vaccinated?	90 (27.3)	240 (72.7)
Do you think the COVID-19 vaccination can protect against COVID-19 infection?	213 (64.5)	117 (35.5)
Does a person still need to practice preventive measures such as wearing a face mask, washing hands, and social distancing after vaccination?	280 (84.8)	50 (15.2)

**Table 3:** Health beliefs and vaccine acceptance.

Variables	Strongly Agree (%)	Agree (%)	Neutral (%)	Disagree (%)	Strongly Disagree (%)
Susceptibility					
There is a possibility of being infected with COVID-19 currently	41 (12.4)	118 (35.8)	104 (31.5)	35 (10.6)	32 (9.7)
I worry about the likelihood of being infected with COVID-19	32 (9.7)	102 (30.9)	109 (33.0)	57 (17.3)	30 (9.1)
The chance of me contacting COVID-19 is high	15 (4.5)	37 (11.2)	112 (33.9)	97 (29.4)	69 (20.9)
I am concerned about those around me being infected with COVID-19	50 (15.2)	91 (27.6)	112 (33.9)	45 (13.6)	32 (9.7)
People could have been infected without symptoms	101 (30.6)	126 (38.2)	66 (20.0)	19 (5.8)	18 (5.5)
Severity					
The complications of COVID-19 are very serious	87 (26.4)	161 (48.8)	59 (17.9)	14 (4.2)	9 (2.7)
I will be very sick if I am infected with COVID-19	32 (9.7)	107 (32.4)	129 (39.1)	42 (12.7)	20 (6.1)
COVID-19 infectivity is very high	62 (18.8)	142 (43.0)	86 (26.1)	28 (8.5)	12 (3.6)
COVID-19 will cause serious damage to my health	55 (16.7)	119 (36.1)	108 (32.7)	31 (9.4)	17 (5.2)
If I catch the disease, I will be threatened with death	26 (7.9)	53 (16.1)	141 (42.7)	72 (21.8)	38 (11.5)
Benefits					
Being vaccinated is a great idea, because it will prevent me from the fear of being infected with COVID-19	71 (21.5)	138 (41.8)	91 (27.6)	21 (6.4)	9 (2.7)
Vaccination reduces my chance of being infected with COVID-19	79 (23.9)	142 (43.0)	82 (24.8)	19 (5.8)	8 (2.4)
The vaccine can prevent COVID-19 complications	62 (18.8)	125 (37.9)	106 (32.1)	27 (8.2)	10 (3.0)
Vaccines prevents diseases effectively	83 (25.2)	145 (43.9)	87 (26.4)	10 (3.0)	5 (1.5)
Vaccinations will allow me to lead my social life in safety	71 (21.5)	118 (35.8)	110 (33.3)	19 (5.8)	12 (3.6)
Barriers					
I worry that my normal life will be interrupted by the side effects of the COVID-19 vaccine	37 (11.2)	84 (25.5)	140 (42.4)	51 (15.5)	18 (55.5)
The COVID-19 vaccine will interrupt my normal life activities	57 (17.3)	136 (41.2)	111 (33.6)	17 (5.2)	9 (2.7)
The COVID-19 vaccine’s safety is a concern to me	65 (19.7)	155 (47.0)	92 (27.9)	12 (3.6)	6 (1.8)
I am concerned about the authenticity of the COVID-19 vaccine	74 (22.4)	123 (37.3)	106 (32.1)	18 (5.5)	9 (2.7)
I am concerned about the affordability of the COVID-19 vaccine	45 (13.3)	87 (26.4)	144 (43.6)	37 (11.2)	17 (5.2)
Cues to action					
If I am given adequate information about the COVID-19 vaccine I will be vaccinated	77 (23.3)	148 (44.8)	77 (23.3)	18 (5.5)	10 (3.0)
I will take the vaccine when someone I know is taking it	28 (8.5)	103 (31.2)	120 (36.4)	54 (16.4)	25 (7.6)
If the government gives free vaccinations, I will be vaccinated	67 (20.3)	101 (30.6)	113 (34.2)	30 (9.1)	19 (5.8)
I have watched media reports promoting COVID-19 vaccinations	61 (18.5)	138 (41.8)	104 (31.5)	19 (5.8)	8 (2.4)
My family, friends, and peers recommend taking the COVID-19 vaccine	44 (13.3)	89 (27.0)	130 (39.4)	51 (15.5)	16 (4.8)
Vaccine acceptance					
I would like to receive the COVID-19 vaccine right away	55 (16.7)	96 (29.1)	126 (38.2)	29 (7.3)	24 (7.3)
I still have some concerns, so I don’t want to receive the COVID-19 vaccine right away	43 (13.0)	61 (18.5)	118 (35.8)	69 (20.9)	39 (11.8)
I’m willing to receive the COVID-19 vaccine even if I’m required to pay for it	37 (11.2)	89 (27.0)	139 (42.1)	38 (11.5)	27 (8.2)
I’m still hesitant about the COVID-19 vaccine	37 (11.2)	64 (19.4)	127 (38.5)	66 (20.0)	36 (10.9)
I’m willing to receive the COVID-19 vaccine no matter what	31 (9.4)	74 (22.4)	141 (42.7)	49 (14.8)	35 (10.6)

**Table 4:** Crosstab showing how vaccine acceptance varies across demographics, health beliefs, and knowledge about the COVID-19 vaccine.

Variable		*N* (%)			*p*-Value
		Total *n* = 330	COVID-19 Vaccine Acceptance		
			Strongly Agree/Agree *n* = 120 (36.4%)	Neutral/Disagree/Strongly Disagree *n* = 210 (63.6%)	
Gender					
	Male	180 (54.5)	68 (37.8)	112 (62.2)	0.319
	Female	150 (45.5)	52 (34.7)	98 (65.3)	
Age					
	25 years and Below	180 (54.5)	63 (35.0)	117 (65.0)	0.326
	26 years and Above	150 (45.5)	57 (38.0)	93 (62.0)	
Residence					
	Currently in China	192 (58.2)	51 (26.6)	141 (73.4)	<0.001
	Not currently in China	138 (41.8)	69 (50.0)	69 (50.0)	
Educational level					
	Undergraduates	172 (52.1)	63 (36.6)	109 (63.4)	0.504
	Graduates and Above	158 (47.9)	57 (36.1)	101 (63.1)	
Program of study					
	Medical-related major	215 (65.2)	88 (40.9)	127 (59.1)	0.012
	Non-Medical related major	115 (34.8)	32 (27.8)	83 (72.2)	
Continent of origin					
	Europe	7 (2.1)	2 (28.6)	5 (71.4)	0.016
	Asia	151 (45.8)	67 (44.4)	84 (55.6)	
	Africa	165 (50.0)	50 (30.3)	115 (69.7)	
	America	3 (1.2)	0 (0.0)	3 (100)	
	Oceania	4 (0.3)	3 (75.0)	1 (25.0)	
Health status					
	Very Good/Good	313 (94.8)	117 (37.4)	196 (62.6)	0.078
	Fair/Bad	17 (5.2)	3 (17.6)	14 (82.4)	
Medical history					
	Diagnosed with a chronic disease	8 (4.2)	2 (57.1)	6 (42.9)	0.400
	Not diagnosed with a chronic disease	322 (95.8)	118 (36.6)	204 (63.4)	
Knowledge					
	Yes	222 (67.2)	97 (43.7)	125 (56.3)	<0.001
	No	108 (32.8)	23 (21.3)	85 (78.7)	
Susceptibility					
	Yes	128 (38.8)	63 (49.2)	65 (50.8)	<0.001
	No	202 (61.2)	57 (28.2)	145 (71.8)	
Severity					
	Yes	168 (50.9)	72 (42.9)	96 (57.1)	0.008
	No	162 (49.1)	48 (29.6)	114 (70.4)	
Benefits					
	Yes	213 (64.5)	107 (50.2)	106 (49.8)	<0.001
	No	117 (35.5)	13 (11.1)	104 (88.9)	
Barriers					
	Yes	193 (58.5)	66 (34.2)	127 (65.8)	0.196
	No	137 (41.5)	54 (39.4)	83 (60.6)	
Cues to action					
	Yes	169 (51.2)	102 (60.4)	67 (39.6)	<0.001
	No	161 (48.8)	18 (11.2)	143 (88.8)	

**Table 5:** Multivariable logistic regression of the factors associated with COVID-19 vaccine acceptance (*N* = 330).

Variable	Sig.	Exp(B)	95% C.I.for EXP(B)	
			Lower	Upper
Gender (ref: Male)	0.708	1.121	0.617	2.036
Age (ref: 25 years and Below)	0.573	0.573	0.560	1.378
Residence (ref: currently in China)	0.07	0.536	0.273	1.052
Educational level (ref: undergraduates)	0.687	1.221	0.461	3.231
Continent of origin (ref: Europe)	0.996			
Asia	0.723	1.877	0.058	60.915
Africa	0.752	1.551	0.102	23.689
America	0.804	1.407	0.095	20.872
Oceania	0.999	0	0	.
Health status (ref: good/very good)	0.62	1.528	0.287	8.143
Medical history (ref: diagnosed with a chronic disease reference)	0.163	3.051	0.637	14.613
Knowledge (ref: No)	0.344	0.724	0.371	1.413
Perceived susceptibility (ref: No)	0.353	1.34	0.723	2.484
Perceived severity (ref: No)	0.678	1.139	0.616	2.107
Perceived benefits (ref: No)	0.002	3.615	1.63	8.018
Perceived barriers (ref: No)	<0.001	0.307	0.159	0.594
Cues to action (ref: No)	<0.001	8.759	4.288	17.89

## Data Availability

The data are available upon reasonable request from the corresponding author. E-mail: youhua98@163.com.

## Supplementary Materials

The following are available online at https://www.mdpi.com/article/10.3390/vaccines9060585/s1, Table S1. The overall scores of the knowledge and beliefs concerning COVID-19 vaccine vaccination.
