# Supplementary material for: Vaccine Acceptance and Its Influencing Factors: An Online Cross-Sectional Study among International College Students Studying in China
Source: Vaccines (Basel). 2021 Jun 2;9(6):585. doi: 10.3390/vaccines9060585 (PMC8228371; doi:10.3390/vaccines9060585)
Supplement: Supplementary file 1 [file vaccines-09-00585-s001.zip › vaccines-1204950-supplementary.pdf]

Table_S1. The overall scores of the knowledge and beliefs concerning COVID-19 vaccine vacinnation

variable	N	(%)
knowledge yes	222	67.2
knowledge No	108	32.8
Susceptible Yes	128	38.8
Susceptible No	202	61.2
severe Yes	168	50.9
severe No	162	49.1
Benefical Yes	213	64.5
Beneficial No	117	35.5
Barrier Yes	193	58.5
not barrier	137	41.5
Cues to action Yes	169	51.2
Cues to action No	161	48.8
